# No Impact of Omega-3 Fatty Acid Supplementation on Symptoms or Hostility Among Patients With Schizophrenia

**DOI:** 10.3389/fpsyt.2020.00312

**Published:** 2020-04-21

**Authors:** Yi Qiao, Cai Ping Liu, Hui Qin Han, Feng Ju Liu, Yang Shao, Bin Xie

**Affiliations:** Shanghai Mental Health Center, School of Medicine, Shanghai Jiao Tong University, Shanghai, China

**Keywords:** schizophrenia, psychopathology, hostility, omega-3, randomized control trial

## Abstract

**Objective:**

This study was aimed to explore the impact of fish oil (Omega-3 fatty acids) on hostility and psychopathology among patients with acute violent schizophrenia.

**Method:**

Sixty seven acute hospitalized patients demonstrating violent behavior in the context of a schizophrenic illness, treated with antipsychotics, were randomly assigned to a supplement with either fish oil (N=32) or placebo (N=35) in a double-blind, placebo-controlled trial. Assessments were conducted at the baseline, week 4 and week 8.

**Results:**

The symptoms and hostility decreased after treatment for 4 and 8 weeks in both groups, with no group differences.

**Conclusions:**

The current study did not find improvements in symptoms or hostility from the Omega-3 fatty acid supplementation in patients with schizophrenia. The implication is that Omega-3 fatty acids do not reduce psychopathology and hostility in acute patients with schizophrenia.

## Introduction

The hypothesis of the membrane phospholipid in schizophrenia is based on Horrobin's postulate (1, 2). Polyunsaturated fatty acids (PUFA), which are regarded as important components of the cell membrane, were found to decline in patients with schizophrenia (3). Lipid dysfunction may be part of the etiology (4, 5). Thus, it was suggested that supplementation of PUFA might improve symptoms of patients with schizophrenia. The mechanism of action of the PUFA is still unclear. Previous studies suggested that PUFAs may interact with the dopamine and serotonin system (4, 6, 7), while some studies implied that PUFAs induced antiapoptotic factors (8, 9).

Some studies testified this hypothesis. Animal studies showed that the administration of omega-3 fatty acids in adolescent rats prevented positive, negative and cognitive symptoms in a ketamine animal model of schizophrenia (10). A study in adolescents and young adults with subthreshold psychosis found that the transition rate to full-threshold psychosis in the Omega-3 PUFA group was much lower as compared to the placebo group. Omega-3 PUFA also significantly reduced positive, negative and general symptoms, alongside improving functioning (4). In first episode psychosis patients, supplementation of the eicosapentaenoic acid (EPA) as an augmented treatment needed 20% lesser antipsychotic medications and had lesser side effects than those treated with antipsychotic medication alone (11). In chronic, severe schizophrenic patients, the EPA supplementation group had significant reduction in their scores for the Positive and Negative Syndrome Scale (PANSS) and that of dyskinesia, than the placebo group (12). However, the results are inconsistent. While there have been several positive studies, a recent meta-analysis showed that Omega-3 had mixed results in patients with stable chronic schizophrenia, with only some patients experiencing significant benefits. Among patients with chronic schizophrenia, use of omega-3 fatty acids by both those experiencing acute exacerbations and those who had discontinued antipsychotic medications resulted in worsening of psychotic symptoms (13). As a consequence, the aim of this study was determine whether supplement PUFA, especially Omega-3 fatty acid PUFA, with acute violent patients could ameliorate symptoms in schizophrenia. We were particularly interested in the symptom of hostility.

Studies in mood disorders (14, 15), borderline personality disorder (16), young males in prison (17); also suggest that PUFAs may influence mood, impulsivity and aggression. Violence is one of the primary reasons for hospitalization currently (18). Blood levels of EPA alone or with docosahexaenoic acid (DHA) have reported being negatively correlated with psychometric measures of aggression (19–21). Dietary supplements of EPA have been associated with a reduction in violent behavior in British and Dutch forensic populations (17, 22). Zanarini's study among borderline personality disorder patients got similar results (16).

Hostility involves unfriendly attitudes including irritability, anger, resentment, or aggression. It can be assessed by PANSS, which defined hostility as a positive symptom (23). A cross-sectional observational study found that consumption of any fish rich in Omega-3 fatty acids, was independently associated with lower odds of high hostility (OR=0.82; 95% CI=0.69–0.97; P=0.02) in young adulthood (24). Some researchers found that concentrations of EPA and DHA in erythrocytes (RBC) showed significant negative correlations with the hostility score of PANSS in acute drug-free schizophrenia (25).

However, to date, there is relatively little research concerning the potential use of Omega-3 fatty acids as a treatment to reduce hostility in acute patients with schizophrenia. This study therefore aimed to explore the impact of Omega-3 fatty acids supplementation on hostility and psychotic symptomatology of acute schizophrenia inpatients.

### Subjects and Methods

Subjects enrolled in this study were patients with an ICD-10 diagnosis of schizophrenia in the Shanghai Mental Health Center from June 2015 to November 2017. This study was approved by the Ethics Committee of the Shanghai Mental Health Center, (Clinic Trial No. NCT02552758). Patients participated in this study during their first 2 weeks of hospitalization. Symptomatology was measured by PANSS. The validity and reliability of the Chinese version of PANSS were acceptable (internal consistency reliability was 0.87, internal consistency reliability of the five dimensions ranged from 0.74 to 0.90) (26). Violent behavior was assessed by the Modified Overt Aggression Scale (MOAS). The MOAS is divided into four subscales: verbal aggression, physical aggression against objects, physical aggression against self and physical aggression against others. The reliability and validity of the Chinese version of the MOAS was modest, with Intra-class correlation coefficient (ICC=0.94, P < 0.001), Kendall's W coefficient of concordance (W=0.83, P= 0.001) and the Mann–Whitney U test (z=− 2.89, P= 0.002) (27). All subjects in this study were required to score higher than 4, which meant that they had severe verbal aggression for 6 months. Assessments were conducted at the baseline, 4 and 8 weeks after treatment. Patients were excluded if they met the criteria for other psychiatric disorders or serious physical diseases. All participants were treated with antipsychotic medication during the study.

Patients were randomly assigned to be treated with either the fish oil, containing 540 mg of EPA and 360 mg of DHA (GNC, USA); or a placebo (10 mg of Vitamin E) once a day, based on a randomization code that was not available to the investigators. The Vitamin E capsule was like the fish oil in color, shape and texture. Participants were unaware about the kind of supplement they received. The nurses were in charge of dispensing the fish oil or placebo to the patients, so that the clinicians and doctors were kept blind to the interventions. The measurement of the levels of DHA and EPA in plasma was taken at the baseline and the 8th week. These levels were analyzed by the gas chromatography-mass spectrometry. All patients or their authorized representatives signed the consent forms.

Chi-square tests, t-test, repeat measure ANOVA, ANCOVA and a correlation analysis were carried out using the Statistical Package for the Social Sciences (SPSS).

## Results

A total of 67 patients (35 males, 32 females) participated in this study. Among them, 32 patients received the intervention of the fish oil and 35 of the placebo. Forty-seven patients (70.1%) completed the 8-week intervention. There were no significant group differences in age, age of first episode of psychosis and the whole duration of illness (**Table 1**). However, gender was not equally distributed between the groups (P=0.036), that is, 21 males and 11 females were allocated in the fish oil group, 14 males and 21 females to the control group. The scores of the MOAS and hostility at the baseline were not statistically different. We documented the psychopharmacologic treatments of both the groups. Since olanzapine and clozapine are most likely to have anti-aggression effects (28, 29), we listed the number of patients who were on these two medications from both groups. There was no significant difference found between the groups (**Table 1**).

**Table 1:** Demographic characteristic of the participants.

	Fish oil group	Placebo group
N	32	35
Male	21	14
Female	11	21
Age	34.09 ± 11.01	34.03 ± 10.38
Age of first episode of schizophrenia	21.44 ± 6.55	22.03 ± 7.27
Duration of illness (year)	12.43 ± 9.92	11.87 ± 8.11
MOAS	14.13 ± 5.63	14.11 ± 6.72
Hostility	4.47 ± 1.54	4.29 ± 1.45
On Clozapine	6	6
On Olanzapine	14	21

MOAS, Modified Overt Aggression Scale.

The plasma concentration of DHA and EPA at the baseline was statistically not different for the two groups. The concentration of EPA increased after 8 weeks of intervention in the fish oil group (p < 0.01), while the concentration of DHA remained unchanged for the two groups.

The scores for the positive scale, negative scale, total scale and hostility of PANSS declined significantly with the treatment going on in both groups at week 4 and week 8, but there were no group differences (**Table 2**). After controlling for gender as a covariate, the result remained same (P=0.524, 0.353, 0.165, and 0.884 for positive scale, negative scale, total scale, and hostility respectively).

**Table 2:** Comparations of positive and negative syndrome scale and hostility between the two groups.

	Fish oil group	Placebo group	Time (F)	time*group (F)
Positive scale (week 0)	26.50 ± 6.38	27.09 ± 5.01		
Positive scale (Week 4)	19.90 ± 5.62	17.94 ± 5.93		
Positive scale (week 8)	17.17 ± 5.52	16.00 ± 6.05	154.25**	0.27
Negative scale (week 0)	20.84 ± 7.63	17.91 ± 6.86		
Negative scale (week 4)	18.37 ± 6.99	14.97 ± 7.43		
Negative scale (week 8)	16.63 ± 6.74	14.65 ± 7.57	21.07**	0.05
Total score (week 0)	91.53 ± 16.75	88.40 ± 13.80		
Total score (week 4)	75.53 ± 16.24	65.44 ± 17.41		
Total score (week 8)	67.38 ± 16.54	59.91 ± 18.67	100.67**	0.19
Hostility (week 0)	4.47 ± 1.54	4.29 ± 1.45		
Hostility (week 4)	3.07 ± 1.39	2.72 ± 1.14		
Hostility (week 8)	2.38 ± 1.06	2.42 ± 1.10	52.30**	0.72

**p < 0.01.

There was a significant correlation between MOAS and hostility at the baseline (R=0.25, P < 0.05). But there was no correlation between hostility and the plasma level of DHA or EPA at the baseline and after treatment.

## Discussion

Scores on the positive, negative and total scale of PANSS declined significantly in both groups at week 4 and week 8, but there were no group differences. This indicated that adjunctive supplementation of Omega-3 fatty acids provided no benefits on symptoms when compared to antipsychotics alone. This result was similar to previous studies among chronic patients with schizophrenia (30–32). One meta-analysis study found that studies in chronic schizophrenia had mixed results, with two studies showing no benefit from EPA, while one study showing that EPA alone led to a worse outcome (2). Even among first episode patients with schizophrenia, one study did not find the extra benefits of EPA on symptom ratings (33). In our study, some patients were not in their first episodes and the average age of the participants was about 34 years. Though all the participants were in the acute phase, some of them were chronic patients with schizophrenia. This may be a confound.

The scores of hostility declined significantly in both groups at week 4 and week 8, but no group differences were found in this study. There was no correlation between hostility and the plasma level of DHA or EPA. In a previous study, significant correlations between hostility and PUFAs in erythrocyte were found in drug-free patients with schizophrenia (25). However, that was a cross-sectional study, so the direction was impossible to determine whether the PUFA supplement could ameliorate hostility in patients with schizophrenia. Another pilot study in which 12-violent treatment-resistant in patients with chronic schizophrenia received a supplement each of DHA, EPA, and vitamin E for 12 weeks and the index of agitation significantly decreased (34). However, it was not an RCT trial; the results might have been confounded by the effects of a pharmacologic treatment. Our study does not support the benefits from Omega-3 fatty acids supplementation on hostility in patients with schizophrenia. Some researchers (35) found that anger stems from the delusion, which in turn mediates the violent behavior in psychotic patients. Violence, aggression and hostility are regarded as symptoms of psychotic patients for some psychiatrists. This result coincides with our first finding that supplements of Omega-3 fatty acids did not show extra benefits on symptoms.

There are several other possible explanations for our negative results: 1) the dosages of DHA and EPA in this study were relatively low (540 mg EPA + 360 mg DHA) and may have been insufficient; 2) our sample size was relatively small, with the possibility of false-negative errors.

There were limitations in this study. First, since gender was not equally distributed among the two groups, we did an analysis of covariance. After controlling for gender as a covariate, the result remained the same. Secondly, we used Vitamin E (10 mg) as a placebo in this study. The reason for the choice of low doses of Vitamin E was due to the shape, texture and color similarities for keeping the blinding. Some studies have employed Vitamin E for the purpose of reducing violence. Gesch etal. (17) found that antisocial behavior in prisons, including violence, was reduced by vitamins, minerals and essential fatty acids. The dosage of Vitamin E was 10 mg in that study, but there were 25 kinds of vitamins and minerals in the tablets. Hence, it was hard to tell how much 10 mg of Vitamin E contributed to the result. Légaré etal. (34) also provided Vitamin E (400 IU) as a supplement together with Omega-3 (400 mg EPA and 200 mg DHA) to patients with schizophrenia and suggested that nutritional supplements might reduce agitation and psychopathology. However, in that study, the dosage of Vitamin E was much higher than in our study and it was not an RCT trial. Hence, the reduction may have resulted from the psychopharmacologic treatment. Though the doses of Vitamin E are low in our study, it is an active substance. Further studies should avoid using an active substance as a placebo to avoid the possible confounding.

Further studies could recruit more patients with schizophrenia who do not commit violent behavior and identify whether the level of Omega-3 PUFA is one of the biomarkers of schizophrenia. Future studies could prolong the intervention of Omega-3 fatty acids, trying to explore the long-term effects on the hostility, violence and psychopathology of schizophrenia.

## Data Availability Statement

All datasets generated for this study are included in the article/supplementary material.

## Ethics Statement

The studies involving human participants were reviewed and approved by the Ethics Committee of Shanghai Mental Health Center. The patients/participants provided their written informed consent to participate in this study.

## Author Contributions

YQ and CL prepared for the manuscript. HH and FL helped to collect samples and do the blood test. YS helped to do the clinic evaluation. BX was responsible for the study proposal.

## Funding

This work was supported by grants from The Three-Year Action Plan for The Construction of Public Health System in Shanghai (GWIV-5, PI: BX), the National Natural Science Foundation of China (81302624, PI: YQ; 81873909, PI: QL), and Quantitative evaluation based strategic research of interventions on relapse of schizophrenia (19411950800, PI: BX).

## Conflict of Interest

The authors declare that the research was conducted in the absence of any commercial or financial relationships that could be construed as a potential conflict of interest.
